# Dry Eye Disease following LASIK, PRK, and LASEK: An Observational Cross-Sectional Study

**DOI:** 10.3390/jcm12113761

**Published:** 2023-05-30

**Authors:** Tal Yahalomi, Asaf Achiron, Roee Arnon, Nir Stanescu, Joseph Pikkel

**Affiliations:** 1 Department of Ophthalmology, Samson Assuta Ashdod Hospital, Faculty of Health Sciences, Ben-Gurion University of the Negev, Ashdod 7747629, Israel; roee.arnon@gmail.com (R.A.); nirstanescu@gmail.com (N.S.); yossefp@assuta.co.il (J.P.); 2 Department of Ophthalmology, Tel Aviv Sourasky Medical Center, Sackler School of Medicine, Tel Aviv University, Tel Aviv 6423906, Israel; achironasaf@gmail.com

**Keywords:** dry eye disease, Dry Eye Workshop severity score (DEWS), LASIK, PRK, LASEK, refractive surgery, ocular surface disease

## Abstract

Dry eye disease is the most frequent non-refractive postoperative complication following refractive surgery. This prospective study investigated the development of dry eye disease after three common refractive laser surgeries: laser in situ keratomileusis (LASIK), photorefractive keratectomy (PRK), and laser-assisted sub-epithelial keratectomy (LASEK). Patients who underwent uneventful refractive surgery in a single private medical center between May 2017 and September 2020 were included. Ocular surface disease was graded according to the Dry Eye Workshop severity (DEWS) classification. Patients were examined 6 months following refractive surgery. The analysis included 251 eyes of 137 patients: 64 eyes (36 patients) after LASEK, 90 eyes (48 patients) after PRK, and 97 eyes (53 patients) after LASIK. At 6 months post-surgery, the DEWS score was higher for the LASIK than the PRK and LASEK groups (*p* = 0.01). For the total cohort, severe DEWS score (grades 3 and 4) at 6 months post-surgery was correlated with female gender (*p* = 0.01) and to the amount of refractive correction (*p* < 0.001), but not to age (*p* = 0.87). In conclusion, LASIK surgery and female gender were associated with dry eye. Patients, particularly those with high myopia, should be counseled about the risk of developing dry eye after refractive surgeries.

## 1. Introduction

Jose Barraquer created the first effective refractive surgery technique in 1963 at the Barraquer Ophthalmologic Clinic in Bogotá, Colombia. His method, known as keratomileusis, or corneal reshaping (from the Greek words kéras, “horn”, and mileusis, “carving”), was developed to correct both myopia and hyperopia [1]. A portion of the cornea is removed and frozen to allow for manual shaping; the modified layer is reinserted back into the eye. Swinger performed the first keratomileusis surgery in the US in 1980 [1].

Refractive surgery remains one of the fastest-evolving areas of ophthalmology, and has the potential to correct high refractive errors. The field has advanced beyond excimer laser surgery in the last ten years. Newer platforms, such as the femtosecond laser, have enhanced the results of conventional techniques and facilitated the development of novel ones, like small incision lenticule extraction [2].

The most prevalent corneal refractive procedures for treating low and moderate myopia are laser in situ keratomileusis (LASIK), laser-assisted sub-epithelial keratectomy (LASEK), and photorefractive keratectomy (PRK) [3]. The degree of ametropia and the patient’s corneal shape are the major factors in selecting the surgical procedure; all the procedures are considered safe and effective. Decision making is also influenced by the patients’ lifestyle, such as involvement in contact sports or vocations with physical activity, by variations in postoperative recovery times, and by ocular surface conditions.

Dry eye disease is considered the most frequent nonrefractive postoperative complication following refractive surgery. The symptoms, including fluctuating vision and a feeling of a foreign body in the eye, decrease quality of life [4]. About 95% of patients have been reported to experience some dry eye symptoms immediately following refractive laser surgery. In comparison, 60% of patients experience dry eye symptoms one month following surgery [5,6]. Post-refractive surgery dry eye often peaks in the first few months following surgery, and symptoms gradually subside for the majority of patients during the year following the surgery. Two independent retrospective studies reported that about 30% of patients sent to tertiary ophthalmology care facilities after receiving LASIK had dry eye disease [7,8]. Although frequently transient, dry eye symptoms are the leading source of patient discontent following corneal refractive surgery [9,10]. Some medical professionals advise photorefractive keratectomy, instead of LASIK or LASEK, as a treatment strategy for patients with ocular surface conditions, yet the evidence until now has been unclear [11,12,13,14,15]. Table 1 summarizes the results of relevant previous studies.

This research aimed to compare the symptoms and severity of dry eye disease after three refractive surgeries.

## 2. Methods

All the data for the study were collected and processed in accordance with the norms and procedures of the Institutional Review Board, as well as the principles described in the Helsinki Declaration.

Consecutive patients who underwent refractive surgery in a single private medical center in Haifa, Israel, between May 2017 and September 2020, were included in this prospective study. We included only young, healthy patients without any ocular history other than myopia (up to −10 diopters) and aged above 18 years. We excluded all the patients with prior ocular diseases or pathologies, including glaucoma, corneal diseases, and prior ocular surgeries; or with systemic diseases such as diabetes, hypertension, and inflammatory or immunologic diseases. We excluded patients who underwent eventful or complicated refractive surgeries.

The sample was stratified into three groups based on surgery type. The selection was non-randomized. All the refractive surgeries in the three groups were performed by one surgeon (J.P.). PRK was preformed using an Allegretto Wave Eye-Q 400 Hz Laser (Alcon Inc., Fort Worth, TX, USA). The corneal epithelium was removed manually. The size of the ablation zone was 6 mm. The LASIK procedure was performed by a BAUSCH & LOMB microkeratome and following ablation with the Allegretto Wave Eye-Q 400 Hz Laser (Alcon Inc., Fort Worth, TX, USA). LASEK was performed using 20% alcohol for 20 s.

Following PRK and LASEK, patients were fitted with a bandage contact lens. All the patients were treated with ofloxacin eye drops (Oflox, Allergan, Dublin, Ireland) four times daily for four days, and with dexamethasone 0.1% eye drops (Sterodex, Fischer pharmaceuticals LTD, Tel Aviv, Israel) four times daily for one month. At postoperative follow-up, the contact lenses were removed after the epithelium was healed, and the Oflox drops were stopped.

Patients were examined and graded for ocular surface disease according to the Dry Eye Workshop severity (DEWS) classification [16], and were examined 6 months following the refractive surgery. Severe DEWS score was defined as grades 3 and 4. This study included only patients with a peri-operative DEWS score of 0. This grading system was selected because of its broad acceptance and ongoing use in recent studies in the field of ocular surface disease [17,18,19,20,21]. The follow-up examinations with DEWS grading were performed by the same surgeon who performed the surgeries (J.P.). Table 2 shows the DEWS scores for the patients.

A three-part categorization system and an updated definition of dry eye were presented in the DEWS classification, created in 2007 to reflect the knowledge of the condition at that time [16]. The updated scoring system comprised nine categories: subjective measures such as patient discomfort and frequency, and severity of complaints; and objective measures. The latter included visual symptoms, conjunctival injection and staining, corneal staining, corneal or tear signs, the appearance of eyelids and meibomian glands, and clinical tests of the ocular surface such as fluorescein tear break-up time and Schirmer score.

### Statistical Analysis

The Kolmogorov–Smirnov test was used to evaluate normal distribution of continuous variables. The chi-square test and Fisher’s exact test were used to compare categorical variables. The Kruskal–Wallis test was used to compare continuous and ordinal variables between categories, and Dunn’s test was applied for post hoc analysis. The Spearman correlation coefficient was used to evaluate associations between continuous and ordinal variables.

All the statistical tests were two-sided and a *p*-value of less than 5% was considered statistically significant. The data were analyzed using the NCSS 2022 Statistical Software (2022) (NCSS, LLC. Kaysville, UT, USA, ncss.com/software/ncss, accessed on 22 March 2023).

Sample size was calculated using a significance level of 5% and power of 80%. The planned ratio of the group size was 1:1.5:1.5, for LASEK, LASIK, and PRK, respectively, as LASEK is performed less commonly. The sample size was calculated to identify at least a moderate difference in the DEWS score between the three procedures (Cohen f = 0.25). Accordingly, at least 160 eyes were needed (40 + 60 + 60 for the three groups).

## 3. Results

In total, the analysis included 251 eyes of 137 patients: 64 eyes (36 patients) after LASEK, 90 eyes (48 patients) after PRK, and 97 eyes (53 patients) after LASIK. Considering all the patients included, the mean age was 27.8 ± 5.6 years. Of the 137 patients, 73 (53.2%) were male. Four patients were excluded from the analysis due to a complicated procedure. Of these, two underwent LASEK: one had an incomplete flap and one had a free flap. Two patients who underwent PRK were excluded from the analysis due to an eccentric treatment. The three groups did not differ according to age (*p* = 0.34), gender distribution (*p* = 0.69), or the mean myopic correction (*p* = 0. 97) (Table 3).

Six months following the LASEK, PRK, and LASIK procedures, the mean DEWS scores were 0.82 ± 0.92, 0.78 ± 0.83, and 1.07 ± 0.86, respectively. The DEWS score was significantly higher 6 months following LASIK than following PRK or LASEK (*p* = 0.01) (Table 4).

Considering all the patients, a severe DEWS score (grades 3 and 4) at 6 months post-surgery was correlated with female gender (*p* = 0.01), but not to age (*p* = 0.87). Moreover, a severe DEWS score was correlated with the mean myopic correction (*p* < 0.001). The correlations of patient characteristics to a severe DEWS score are summarized in Table 4. Among patients who underwent LASEK, a severe DEWS score was correlated with the mean myopia (*p* = 0.05) and to female gender (*p* = 0.02), but not to age (*p* = 0.60). Among patients who underwent PRK, a severe DEWS score was not correlated with any of the parameters examined. Among patients who underwent LASIK, a severe DEWS score was correlated with the amount of refractive correction (*p* = 0.001) and to female gender (*p* = 0.04) (Table 4).

## 4. Discussion

To our knowledge, this is the first study to compare dry eye symptoms after LASIK, LASEK, and PRK. We found a significantly increased risk of dry eye disease after LASIK than after the other procedures, as reflected in the higher mean DEWS score 6 months after surgery.

The significance of this research is that complications from refractive surgery still include ocular surface disease as their most common cause [13]. As patients’ visual comfort and quality of life directly correlate with the degree of dry eye disease, post-refractive dry eye illness should be addressed properly [16].

Our findings support a prospective study by Lee et al. of 36 eyes following PRK and 39 eyes following LASIK [12]. Tear production was more reduced 6 months after LASIK than after PRK.

In our study, the patients who underwent the three procedures did not differ in age, gender, or the amount of refractive correction. This contrasts with a previous study that reported baseline differences between patients who underwent the compared procedures [15].

Kim et al. reported that lamellar cutting of the cornea during LASIK affects corneal sensitivity, and noted that the cornea did not recover to its pre-operative level even after 6 months [22]. This could explain the greater severity of dry eye disease we observed following LASIK compared to other surgeries. A number of studies showed that corneal sensitivity decreased after PRK but rebounded to nearly normal levels after 3 months [23,24,25]. These findings could perhaps explain the potential of more rapid corneal re-innervation following PRK and LASEK than following LASIK, and could explain our results. Pe’rez-Santonja et al. found that corneal sensitivity in the ablated zone in the context of mild myopia is more decreased following LASIK than PRK, over the first 3 months post-surgery [26]. Corneal sensitivity values following the two procedures were only comparable after 6 months. In a study by Lee et al., tear secretion and tear film stability were less at 3 months after LASIK than after PRK [12]. That study showed reduced tear secretion and tear film stability 6 months after LASIK, yet without statistical significance. These indices did not return to their pre-operative levels. In LASIK, the nerves of the central cornea are cut by the microkeratome, in addition to the laser ablation for myopia correction. Due to decreased ocular sensation, tear production could be decreased, and this could lead to increased tear osmolarity. Not previously having worn eyeglasses may cause an increase in tear film evaporation, which in turn may cause an increase in tear osmolarity.

In contrast to the above, some studies found no difference between procedures in postoperative dry eye symptoms (Table 1). However, none of the studies compared the three surgeries examined herein, and all the previous studies included fewer patients. According to Kanellopoulos et al., corneal sensation was much better following LASIK than PRK [27]. Sauvageot et al. conducted a prospective comparative observational study of 44 eyes with myopia: 22 eyes treated by LASIK and 22 treated by PRK [11]. After one year, the ocular surface conditions following both procedures were regarded as clinically unmodified and even. Likewise, in a study by Murakam et al., dry eye symptoms and intensity, visual changes, and foreign body sensation were increased above baseline in the early postoperative period of both LASIK and PRK [14]. All dry eye, fluctuating vision, and foreign body sensation symptoms subsided to their pre-operative baseline levels within one year after surgery. The single study that compared LASIK and LASEK reported no significant differences in dry-eye disease markers or in tear osmolarity at any stage after surgery, up to 1 year [15].

As expected, we found a correlation between a severe DEWS score and the amount of refractive correction. This is in accordance with a previous study [9]. The explanation of the correlation with ocular surface disease is that the greater the amount of myopia that is treated, the greater the required ablation depth. The corneal sensory nerves enter the peripheral cornea mid-stromal depth and run centrally, and then branch off anteriorly into the corneal epithelium, where free nerve terminals are found [28]. Greater depth of laser ablation necessitates greater distance of the surgically cut nerve trunks, to regrow sufficiently such as to re-nerve the corneal epithelium after surgery.

In an analysis that included all our patients, female gender was correlated with a more severe score of dry eye. This relation was maintained in separate analyses of patients who underwent LASEK and LASIK. Previous retrospective studies [6,29] found an association of female gender with chronic tear dysfunction following LASIK surgery. The greater risk of dry eyes in women has been linked to decreased levels of androgen hormones, which support tear secretion by the lacrimal glands [30]. Notably, a prospective study [9] found no association of dry eye severity with gender. Our finding that age was not an important risk factor for ocular surface disease following LASIK corroborates other studies [9,29].

Our study has several limitations. First, the number of eyes was 251, and a larger number of patients should be included in further studies, as well as more refractive laser procedures, and multiple medical facilities. Future research is required to confirm the validity and replicability of those conclusions.

Secondly, the maximum follow-up was 6 months, and long-term implications should be further examined and tested. Thirdly, the inevitable selection criteria for each procedure are a limitation of this study, which did not include randomization. Peri-operative DEWS scores were not collected; although this is similar to previous studies on this subject, it represents a limitation in our study. Additionally, this study utilized binocular data, though earlier studies had also presented this type of data analysis [12,14].

Further, in the LASIK procedure, a manually operated microkeratome was used, rather than the more recently developed femtosecond flap formation method.

Our findings suggest that careful monitoring and appropriate dry eye treatment are essential, especially in the early and late postoperative phases of LASIK. Additional factors that may impact tear secretion, though not explored in this study, should be examined. Careful selection of the best surgery for each patient is important. Possibly, LASIK may not be the best option for candidates for surgery who are at risk of developing a dry eye condition, such as programmers, or those who spend considerable time in front of screens.

In summary, in this prospective large-scale study, dry eye disease was more common following LASIK than other procedures. Female gender was also associated with dry eye. Patients, particularly those with high myopia, should be counseled about the risk of developing dry eye after refractive surgeries.

## Figures and Tables

**Table 1:** Previous studies that compared dry eye symptoms following refractive laser surgeries.

Author	Year	Region	Number of Eyes	Study Method	Type of Procedures	Follow Up [Months]	Outcomes
Lee JB [12]	2000	Korea	75	Prospective study	PRK vs. LASIK	3 and 6	The decrease in tear secretion was greater following LASIK surgery than after PRK at 6 months.
Dooley [15]	2012	Ireland	85	Prospective controlled cross-sectional observation study	LASIK vs. LASEK	3, 6, and 12	At any point up to 1 year post-surgery, changes were not discerned between patients who underwent LASIK and LASEK, in markers of dry-eye disease or tear osmolarity.
Murakami [14]	2012	USA	68	Prospective randomized clinical trial	LASIK vs PRK	1, 3, 6, and 12	In the early period after LASIK and PRK, dry eye symptoms and severity were increased compared to baseline. However, the symptoms subsided to pre-operative levels by 1 year after surgery.
Denoyer [13]	2014	France	60	Prospective, comparative study	SMILE vs. LASIK	1 and 6	One month following surgery, both groups had high rates of mild to moderate dry eye disease, which remained considerably higher in the LASIK than the SMILE group six months later.
Sauvageot [11]	2017	Spain	44	Prospective comparative observational study.	LASIK vs. PRK	3, 6, and 12	After 3 months, the only difference between the two groups was lower corneal sensitivity in the LASIK group. After 1 year, the ocular surface conditions of both groups were regarded as clinically unmodified.

PRK: photorefractive keratectomy; LASIK: laser-assisted in situ keratomileusis; SMILE: small incision lenticule extraction.

**Table 2:** Dry Eye Workshop severity (DEWS) scale [16].

	Severity Grade				
Parameter	0	1	2	3	4
Schirmer test (mm/5 min)	35	7	5	2	0
TBUT (seconds)	45	7	5	3	0
Staining (NEI scale)	0	3	8	12	20
OSDI (%)	0	15	30	45	100
Meibomian grading	0	1	2	3	4
Osmolarity (mOsm/L)	275	308	324	364	400

Patients were examined and graded for ocular surface disease according to the DEWS classification for dry eye [16] and were examined 6 months following the refractive surgery. First introduced in the Subcommittee of the International Dry Eye Work Shop in 2007, the DEWS score consists of patients’ complaints of discomfort, severity and frequency, visual symptoms, conjunctival injection and staining, corneal staining (severity/location), corneal/tear signs, lid/meibomian glands, fluorescein tear break-up time (s), and Schirmer score [16]. TBUT: tear breakup time; NEI: National Eye Institute; OSDI: Ocular Surface Disease Index.

**Table 3:** Baseline peri-operative characteristics and DEWS scores according to refractive surgeries: LASEK, PRK, and LASIK.

	LASEK	PRK	LASIK	*p* Value
Number of eyes (patients)	64 (36)	90 (48)	97 (53)	
Mean age years	27.5± 5.88	27.3 ± 5.40	28.4 ± 2.09	0.34 #
Male gender	55.3%	51.1%	49.4%	0.69 *
Mean myopic correction [D]	6.0 ± 2.23	6.2 ± 2.03	6.2 ± 2.09	0.97 #
DEWS score	0.82 ± 0.92	0.78 ± 0.83	1.07 ± 0.86	0.01 #

# Kruskal–Wallis test; * Chi square. DEWS, Dry Eye Workshop severity scale; LASEK, laser-assisted sub-epithelial keratectomy; PRK, photorefractive keratectomy; LASIK, laser in situ keratomileusis.

**Table 4:** Correlations of patient characteristics to severe DEWS scores (score 3 or 4), at 6 months following LASEK, PRK, and LASIK.

	LASEK	PRK	LASIK	Statistical Test Applied
Number of eyes (patients)	64 (36)	90 (48)	97 (53)	
Age	*p* = 0.60	*p* = 0.32	*p* = 0.80	Spearman Rank Correlation Test
Female gender	*p* = 0.02	*p* = 0.72	*p* = 0.04	Mann–Whitney U
Myopia correction [D]	*p* = 0.05	*p* = 0.26	*p* = 0.001	Spearman Rank Correlation Test

LASEK, laser-assisted sub-epithelial keratectomy; PRK, photorefractive keratectomy; LASIK, laser in situ keratomileusis.

## Data Availability

Data is unavailable due to privacy or ethical restrictions.

## References

[1] Azar D.T. (2007). Laser and mechanical microkeratome. Refractive Surgery.

[2] Jabbour S., Bower K.S. (2021). Refractive Surgery in the US in 2021. JAMA Insights.

[3] Shortt A.J., Allan B.D.S., Evans J.R. (2013). Laser-assisted in-situ keratomileusis (LASIK) versus photorefractive keratectomy (PRK) for myopia. Cochrane Database Syst. Rev..

[4] Raoof D., Pineda R. (2014). Dry Eye after Laser In-Situ Keratomileusis. Semin. Ophthalmol..

[5] Hovanesian J.A., Shah S.S., Maloney R.K. (2001). Symptoms of dry eye and recurrent erosion syndrome after refractive surgery. J. Cataract Refract. Surg..

[6] Albietz J.M., Lenton L.M., McLennan S.G. (2002). Effect of laser in situ keratomileusis for hyperopia on tear film and ocular surface. J. Refract. Surg..

[7] Levinson B.A., Rapuano C.J., Cohen E.J., Hammersmith K.M., Ayres B.D., Laibson P.R. (2008). Referrals to the Wills Eye Institute Cornea Service after laser in situ keratomileusis: Reasons for patient dissatisfaction. J. Cataract Refract. Surg..

[8] Jabbur N.S., Sakatani K., O’Brien T.P. (2004). Survey of complications and recommendations for management in dissatisfied patients seeking a consultation after refractive surgery. J. Cataract Refract. Surg..

[9] De Paiva C.S., Chen Z., Koch D.D., Hamill M.B., Manuel F.K., Hassan S.S., Wilhelmus K.R., Pflugfelder S.C. (2006). The Incidence and Risk Factors for Developing Dry Eye After Myopic LASIK. Am. J. Ophthalmol..

[10] Nettune G.R., Pflugfelder S.C. (2010). Post-LASIK Tear Dysfunction and Dysesthesia. Ocul. Surf..

[11] Sauvageot P., Julio G., Alvarez de Toledo J., Charoenrook V., Barraquer R.I. (2017). Femtosecond laser-assisted laser in situ keratomileusis versus photorefractive keratectomy: Effect on ocular surface condition. J. Cataract Refract. Surg..

[12] Lee J.B., Ryu C.H., Kim J.H., Kim E.K., Kim H.B. (2000). Comparison of tear secretion and tear film instability after photorefractive keratectomy and laser in situ keratomileusis. J. Cataract Refract. Surg..

[13] Denoyer A., Landman E., Trinh L., Faure J.F., Auclin F., Baudouin C. (2015). Dry eye disease after refractive surgery: Comparative outcomes of small incision lenticule extraction versus LASIK. Ophthalmology.

[14] Murakami Y., Manche E.E. (2012). Prospective, Randomized Comparison of Self-reported Postoperative Dry Eye and Visual Fluctuation in LASIK and Photorefractive Keratectomy. Ophthalmology.

[15] Dooley I., D’Arcy F., O’Keefe M. (2012). Comparison of dry-eye disease severity after laser in situ keratomileusis and laser-assisted subepithelial keratectomy. J. Cataract Refract. Surg..

[16] Foulks G. (2007). The Definition and Classification of Dry Eye Disease: Report of the Definition and Classification Subcommittee of the International Dry Eye WorkShop (2007). Ocul. Surf..

[17] Mangoli M.V., Bubanale S.C., Bhagyajyothi B.K., Goyal D. (2023). Dry eye disease in diabetics versus non-diabetics: Associating dry eye severity with diabetic retinopathy and corneal nerve sensitivity. Indian J. Ophthalmol..

[18] Bhatt K., Singh S., Singh K., Kumar S., Dwivedi K. (2023). Prevalence of dry eye, its categorization (Dry Eye Workshop II), and pathological correlation: A tertiary care study. Indian J. Ophthalmol..

[19] Vidal-Rohr M., Craig J.P., Davies L.N., Wolffsohn J.S. (2023). The epidemiology of dry eye disease in the UK: The Aston dry eye study. Cont. Lens Anterior Eye.

[20] Nilsen C., Graae Jensen P., Gundersen M., Utheim Ø.A., Gjerdrum B., Gundersen K.G., Jahanlu D., Potvin R. (2023). The Significance of Inter-Eye Osmolarity Difference in Dry Eye Diagnostics. Clin. Ophthalmol..

[21] Gouws P., Barabas S., Gouws A. (2023). Efficacy of Portable 445 nm Laser Versus Intense Pulsed Light Treatment for Dry Eye: A Prospective Randomized Pilot Study. Photobiomodulation Photomed. Laser Surg..

[22] Montés-Micó R., Charman W.N. (2001). Intraocular pressure after excimer laser myopic refractive surgery. Ophthalmic Physiol. Opt..

[23] Kohlhaas M., Spoerl E., Boehm A.G., Pollack K. (2006). A correction formula for the real intraocular pressure after LASIK for the correction of myopic astigmatism. J. Refract. Surg..

[24] Ishikawa T., Park S.B., Cox C., del Cerro M., Aquavella J.V. (1994). Corneal sensation following excimer laser photorefractive keratectomy in humans. J. Refract. Corneal Surg..

[25] Campos M., Hertzog L., Garbus J.J., McDonnell P.J. (1992). Corneal sensitivity after photorefractive keratectomy. Am. J. Ophthalmol..

[26] Pérez-Santonja J.J., Sakla H.F., Cardona C., Chipont E., Alió J.L. (1999). Corneal sensitivity after photorefractive keratectomy and laser in situ keratomileusis for low myopia. Am. J. Ophthalmol..

[27] Kanellopoulos A.J., Pallikaris I.G., Donnenfeld E.D., Detorakis S., Koufala K., Perry H.D. (1997). Comparison of corneal sensation following photorefractive keratectomy and laser in situ keratomileusis. J. Cataract Refract. Surg..

[28] Belmonte C., Aracil A., Acosta M.C., Luna C., Gallar J. (2004). Nerves and sensations from the eye surface. Ocul. Surf..

[29] Konomi K., Chen L.L., Tarko R.S., Scally A., Schaumberg D.A., Azar D., Dartt D.A. (2008). Preoperative characteristics and a potential mechanism of chronic dry eye after LASIK. Investig. Ophthalmol. Vis. Sci..

[30] Smith J.A., Vitale S., Reed G.F., Grieshaber S.A., Goodman L.A., Vanderhoof V.H., Calis K.A., Nelson L.M. (2004). Dry eye signs and symptoms in women with premature ovarian failure. Arch. Ophthalmol..

